# The Effects of County Public Hospital Reform on the Consumption and Costs of Antibiotics: Evidence from a Quasinatural Experiment in Jiangsu, China

**DOI:** 10.1155/2020/9262170

**Published:** 2020-10-22

**Authors:** Ying Wang, Yulei Zhu, Xiaoliang Liu, Xinglu Xu, Wenqing Fang, Xin Li

**Affiliations:** ^1^Department of Health Policy, School of Health Policy and Management, Nanjing Medical University, Nanjing 211166, China; ^2^Department of Infection Management, Jiangyin People's Hospital, Jiangyin 214400, China; ^3^Department of Clinical Pharmacy, School of Pharmacy, Nanjing Medical University, Nanjing 211166, China; ^4^Center for Global Health, School of Public Health, Nanjing Medical University, Nanjing 211166, China

## Abstract

**Background:**

Overuse of antibiotics is a major driver for rapid spread of antimicrobial resistance worldwide, particularly common in China. The close linkage between hospital revenue and sales of drugs has become the key incentive for overprescription of antibiotics. Since 2009, the Chinese government implemented a series of measures to cut off the link, including removing the markup of drugs, increasing financial subsidies, and adjusting charges for medical service.

**Objective:**

To evaluate the impacts of county public hospital reform on the consumption and costs of procured antibiotics in Jiangsu province.

**Methods:**

A quasiexperiment design was conducted in Jiangsu province where 99 county public hospitals implemented the reform successively in different periods. Of these, 37 county public hospitals implemented the reform since January 2013, which were regarded as the intervention group, and the remaining 62 hospitals were included in the control group. A difference-in-differences (DID) analysis with generalized linear regressions was used on the procurement records of antibiotics from January 2012 to December 2013. Modified Park test was used for family distribution and Box–Cox test for log link. Placebo tests were employed to test the common-trend hypothesis of two groups.

**Results:**

For the intervention group, the average volume of procured restricted antibiotics and injectable antibiotics increased by 24.12% and 2.75% while the costs increased by 19.01% and 9.09%, respectively. The average costs per DDD of restricted and injectable antibiotics were much higher than unrestricted and oral antibiotics. The DID results showed that the reform had a positive impact on the average volume (*p* = 0.005) and costs (*p* = 0.001) of nonrestricted antibiotics. In addition, the implementation of the reform was associated with a reduction in volume (*p* = 0.031) and costs (*p* = 0.043) of procured oral antibiotics. The reform also contributed to an increase in average costs per DDD of total antibiotics (*p* = 0.049).

**Conclusions:**

The reform is effective in reducing the consumption and costs of unrestricted and oral antibiotics, but it has failed to reduce the consumption and costs of expensive restricted and injectable antibiotics, leading to increased burden of diseases. It is critical that the health policy initiatives can deincentivize overuse of antibiotics at both hospital and individual physician's levels. The reform should enforce government financial support, improve hospital governance, optimize performance evaluation, and establish specialized management approach for antibiotic use.

## 1. Introduction

Antibiotics are widely used for prevention and treatment of bacterial infectious diseases. Overuse of antibiotics is a key driver for rapid spread of antimicrobial resistance (AMR), a global public health crisis [1]. AMR has resulted in the prolonged duration of treatment, increased healthcare costs, and increased morbidity and mortality of bacterial infection [2–4]. Aiming at addressing the issue of rapid spread of AMR, WHO developed a series of strategies to contain excessive use of antibiotics; however, the consumption of antibiotics is still on rise across the world [5].

China has the most rapid growth of AMR all over the world, and the overuse of antibiotics is more serious due to overprescribing of medications [6, 7]. For instance, the prescription rate of antibiotics for inpatients and outpatients is high in China [7–9]. It is estimated that the hospitalized patients using the antibiotics accounted for 50.3% of total inpatients and the outpatients involving an antibiotic prescription ranged from 41% to 60% [10–12]. In addition, the consumption of antibiotics is common in hospitals and primary healthcare institutions. Compared with most European countries, the consumption of fluoroquinolones and third- and fourth-generation cephalosporins per capita was higher in tertiary hospitals in China [13]. An empirical study showed that the primary healthcare institutions consumed 80% of total antibiotics in Shandong [14]. Furthermore, it is estimated that the direct economic loss due to the overuse of antibiotics ranged from 92.55 to 98.93 billion RMB and the indirect economic loss was from 17.37 billion to 18.12 billion yuan in 2015 [15].

It is commonly recognized that the root of overprescription of antibiotics in China is the distorted pricing schedule propelled by the market-oriented economic reforms in the 1980s [16]. The government changed the financial subsidy system for public hospitals in the transition period of economic reform, which led to a reduction in the state financing. During the period of planned economy, 50%-60% of the gross revenues of public hospitals sourced from the financial subsidy of government, whereas it fell to 11% during the period of market economy [17]. The public hospitals were required to balance the income from drugs and health service, medical costs, and financial subsidies. In 1989, in order to offset the decrease of financial subsidies, the China State Council permitted a markup of 15% on the sales on pharmaceutical products for public hospitals [18]. However, the fees of medical service still were kept low. Therefore, for the purpose of profit-seeking, public hospitals were encouraged to shift emphasis from medical services to pharmaceutical products by adding unnecessary prescriptions [19].

Moreover, in order to give greater autonomy to public hospitals and establish market competitions, the government instituted the policy to allow hospitals to manage residual revenue for personnel bonuses. Although the baseline salaries of hospital employees were relatively low, evidence demonstrated that the average bonus of hospital employees accounted for about 30%-40% of total salary they bring in [20, 21]. The close linkage between bonus and hospital revenue had become the key incentive for hospital employees to overprescribe medications based on the profit rather than cost-efficacy [22]. For example, an empirical study indicated that 98% outpatients with a common cold were prescribed with antibiotics. Evidence also reported that respiratory tract infections are the reason for 78% of all antibiotic prescribing in public hospitals, which far exceeded the WHO recommendation and constituted a significant cost to the health insurance programs [23, 24].

Such prescription patterns indicated the prevalent use of antibiotics, and the economic incentives for hospital employees were the main cause of antibiotics abuse [25, 26]. In turn, the distorted pricing schedule also gave an opportunity for pharmaceutical companies to increase the price, promote more expensive drugs, and encourage unnecessary prescriptions. As a result, the phenomenon that physicians received kickbacks from pharmaceutical salesman was prevalent [27], which led to the shortage of low-price drugs. Antibiotics became most commonly prescribed for patients in healthcare institutions at all levels in China [26]. Since 2000s, the government made various efforts to curb climbing drug prices and reduce overuse of medicines, including fixing the upper limit price of antibiotics and other targeted drugs and controlling the proportion of drug sales of the total revenues in hospitals [4, 28, 29]. Unexpectedly, these efforts made limited progress in the overuse of drugs. The hospital revenues were mainly sourced from drug sales and the proportion of drug costs was reaching 42.7% of total health expenses in 2008, while in Organisation for Economic Co-operation and Development (OCED) countries, the drug costs only accounted for 17% of total health spending [30]. The maximum level of antibiotic prescribing rate was 30% recommended by WHO whereas the rate in China was 80% [10]. Additionally, the per capita use of antibiotics is still in a higher level than the recommended [31].

In 2009, with the purpose of improvement in rational use of medicines and accessibility to drugs and health services, China carried out a new round of healthcare system reforms. Public hospital reform, as one of the key components of the healthcare reform, was established to develop modern hospital management system and hierarchical diagnosis and treatment system. The Chinese government put an emphasis on comprehensive county public hospital reforms, and 311 county public hospitals were selected as the pilot hospitals. A county public hospital, which is a medical center in county areas, provides healthcare for urban and rural patients and can play a leading role in three-tier healthcare network. It is reported that 11722 county public hospitals accounted for 47.44% of all the hospitals [32] and more than 900 million people had accessibility to health services provided by county public hospitals in China [33]. The main measures of the public hospital reform included improving the financial reimbursement mechanism and restructuring the management system, as well as promoting the tendency for public welfare of public hospitals [34]. Zero-markup policy for drug sales was an important measure for compensation system reform of public hospitals which was first implemented in primary healthcare institutions. Previous studies indicated that the zero-markup reform could exert a positive impact on decreasing the income of drug sales in primary healthcare institutions [35, 36]. Under the background of zero-markup reform for drug sales, primary healthcare institutions reduced the cost of patients' drug spending. Meanwhile, a provincial tendering platform and supply chain system was established for the procurement of medicines [37].

As the success of reform became evident in the primary healthcare institutions, it was gradually extended to the public hospitals. The purpose of zero-markup reform for drug sales was to completely eradicate supply-induced demand for drugs. However, due to different financial subsidy policies, public hospitals have gradually shifted from “supply-induced demand for drugs” to “supply-induced demand for services” [38]. Unlike primary care institutions, public hospitals were only partially funded by government financial subsidy. To compensate for the loss of drug sales, they had to increase the charge standard on medical service and add unnecessary medical examination and overtreatments [35, 36].

As public hospitals were removed from the profits from the drugs, it raised concerns that the prescription patterns might be changed after the implementation of public hospital reforms. Recently, many studies focused on the effects of county public hospital reform in China. Mao and Chen [37] demonstrated that the implementation of zero-markup policy resulted in a reduction in the purchasing prices of drugs in the list effectively. Zhang et al. [39] indicated that the county public hospital reform contributed to an increase of 385 RMB in out-of-pocket expenses and a reduction of 147 RMB in inpatient medication spending. A study in Guangxi discovered that the county public hospital reform had not improved the operating efficiency of hospitals and clinical terminal quality effectively [40]. Generally, a number of studies have focused on the effects of county public hospital reform on price and costs of medicines, healthcare quality, and the operating efficiency. Only one study evaluated the impact of the county public hospital reform on procurement volume and cost of antibiotics in Hubei Province, Central China [16]. The study demonstrated that county public hospital reform actually led to an increased total cost of antibiotics due to compensation for the loss of income from the drug sales [16]. However, the findings of the impacts of reform on county public hospitals in different regions have been mixed. For instance, Wang et al. [41] found the efficiency of county public hospitals in Central China was lower than that in Eastern and Western China.

Compared with nonantibiotic drugs, antibiotics are more likely to be used inappropriately in all clinical settings. For instance, a cross-sectional study in China found that the broad-spectrum antibiotics were commonly prescribed to patients without clear indications of bacterial infections [42]. However, some other commonly abused drugs, such as proton-pump inhibitor, are used in patients with specialized diseases. In other words, antibiotics are being used on a much larger scale than other commonly abused drugs. The same as all of the medicines, the inappropriate use of antibiotics can be ascribed to the economic incentives that originate from the healthcare system in China. Therefore, in order to better measure the effects of county public hospital reform on the overuse of drugs in public hospitals comprehensively, we selected antibiotics as the study sample. This study is aimed at evaluating the impacts of county public hospital reforms on the consumption and costs of antibiotics in Jiangsu province, Eastern China.

## 2. Materials and Methods

### 2.1. Settings

The study was conducted in Jiangsu province, located in the Yangtze River Delta in Eastern China with a population of 79.6 million in 2014 and an area of 107,200 km^2^. The annual average income per capita in Jiangsu was 27173 RMB in 2014, ranking in the top range of all provinces and municipalities. Jiangsu has more than 30000 healthcare institutions, and healthcare is primarily provided by 178,600 licensed physicians and 188,800 registered nurses. Approximately 22.4 physicians and 45.8 hospital beds for every ten thousand people are available in Jiangsu [43].

### 2.2. The County Public Hospital Reform in Jiangsu

In response to the call for county public hospital reform from the central government, the government of Jiangsu province selected 16 pilot counties and started the reform in January 2013 initially. According to the policy document issued by the government of Jiangsu province in 2012, the main goal of this reform was to promote the reimbursement mechanism of county public hospitals [44]. Specifically, five measures were included: (1) removing the 15% markup on the drug sales from wholesale to retail in county public hospitals; (2) adjusting prices of medical service, such as charging higher prices for service requiring professional skills and charging lower prices for examinations; county public hospitals followed the reformed fee schedules, which led to an increase in service fee of nursing, diagnostic, and surgical procedures and a decrease in service fee of medical examinations; (3) increasing the financial subsidies for the public hospitals: to guarantee the successful implementation of zero markup on the sale of medicines, the government increased financial subsidies to hospitals to compensate for their income loss; the total amount of the subsidies to county public hospitals should be determined by the markup on the drug sales; (4) improving payment reform of health insurance: a purely fee-for-service (FFS) system was transferred to a mixed payment system that included prospective payment methods such as capitation and diagnostic-related groups (DRG); for instance, under DRG payments, the hospitals receive a prospectively set fixed amount for each admission according to its DRG; the hospitals can keep any savings but also bear financial risk of any cost overrun for hospital patients [45]; (5) improving the drug centralized procurement system. However, the drug centralized procurement system has already been implemented since 2001 in Jiangsu province. Furthermore, no detailed and exercisable guidelines on drug centralized procurement reform in this county public hospital reform were provided. Therefore, the drug centralized procurement system was excluded from the key measures. The intention of county public hospital reform was to completely eradicate the perverse financial incentives for the overuse of drugs. All of the four key measures were targeted to cut off the economic incentives in county public hospitals (Table 1).

According to the guidelines for price adjustment of medical services in county-level public hospital comprehensive reform pilot hospitals in Jiangsu province, the amount of the financial subsidies to county public hospitals was determined by removing the markup on the drug sales [35]. Usually, Jiangsu province is divided into three regions based on the economic status and geographic position: northern region (low income), central region (medium income), and southern region (high income). It should take into account the economic development levels of different regions, their actual situations, and affordability of the governmental fiscal capacity. In the northern region, the government subsidies for the pilot county public hospitals account for 5% of share of removing the markup on the drug sales. In the central region, the government subsidies for the pilot county public hospitals account for 10% of share of removing the markup on the drug sales. In the southern region, the government subsidies for the pilot county public hospitals account for 20% of share of removing the markup on the drug sales [35].

The pilot county public hospitals were required to implement the reform, and the nonpilot public hospitals were encouraged to explore effective measures or continued to implement the old measures [46].

### 2.3. Design

A quasiexperimental difference-in-differences design was employed to evaluate the impacts of county public hospital reform on the consumption and costs of antibiotics. There are 45 counties in Jiangsu, and each has one general public hospital and several hospitals specializing in traditional Chinese medicine, maternity and child health, and others. At the initial stage of the implementation of county public hospital reform in Jiangsu in January 2013, 37 public hospitals of 16 counties were selected to implement the reform, and the remaining 62 public hospitals of the other 29 counties joined in January 2014. Therefore, the 37 public hospitals that implemented the reform from January 2013 to December 2013 were regarded as the intervention group while the 62 public hospitals which have not implemented the reform during January 2013 to December 2013 were the control group. The entire study period was from January 2012 to December 2014. The characteristics of 45 counties in which the hospitals included in the study are located are shown in Table 2.

### 2.4. Data Collection and Measurement

Since 2009, the procurement database has been integrated in the Hospital Information System (HIS) of public hospitals in Jiangsu province. The HIS database not only captures the type and volume of drugs purchased by the hospital pharmacy but also records the information on drugs in stock, prescribed, and dispensed. The electronic procurement records of antibiotics were extracted from the HIS of 99 county public hospitals, which resulted in 324,693 procurement records over the 36-month period from January 2012 to December 2014 totally. Monthly data were collected, which included the following variables: healthcare institution, unique chemical substance name, generic name, unit strength, pack size, dosage form, and costs. In August 2012, the guideline for antibiotic stewardship in China, *the Administrative Measures for Clinical Use of Antimicrobial Agents*, was formally issued by the National Health Commission of P.R. China. In the guideline for antibiotic stewardship, antibiotics were categorized into nonrestricted, restricted, and controlled antibiotics. Accordingly, the antibiotics were categorized into three subgroups: nonrestricted, restricted, and controlled antibiotics [47]. Furthermore, they were classified into oral and injectable antibiotics based on the administration route.

Three outcome indicators were used to evaluate the impacts of county public hospital reform on the procurement of all antibiotics and the different subgroups: the average volume of procured antibiotics, the average costs of procured antibiotics, and the average costs per DDD of procured antibiotics.

The consumption of procured antibiotics was evaluated by average volume (DDDs/1000, defined daily dose) of procured antibiotics per hospital per month.

(1) $$\begin{array}{c} \text{Average volume }\left(\frac{\text{DDDs}}{1000}\right)=\frac{\text{Total DDDs}}{\text{number of hospitals}\times 12\text{ months}}. \end{array}$$

The DDD at product level (DPP = unit strength∗pack size/DDD) was calculated in accordance with the ATC template [47]. Then, the DDDs were calculated where DDD per package is multiplied by the corresponding number of procured antibiotics.

The costs of procured antibiotics were measured by the average costs and average costs per DDD of antibiotics. The average costs of antibiotics based on the price and purchase volume were calculated by using total costs per month divided by the number of hospitals. The average costs per DDD of procured antibiotics were estimated by average costs of antibiotics divided by average volume of corresponding antibiotics. With reference to the WHO definition of expenditure per DDD, the indicator “average costs per DDD” represents the actual cost paid by a health system for specific medicines [48]. The indicator “average costs per DDD” could also be regarded as the unit price for drugs. For instance, the similar indicators, such as “costs per DDD” and “average unit price per DDD,” were used in some drug utilization studies [16, 49]. If a hospital was found to have higher average costs per DDD than another hospital, it indicated that the drugs in the hospital were much more expensive than another hospital. Provided that the patients' daily income kept unchanged, higher average costs per DDD could lead to higher burden of disease. Therefore, the indicator “average costs per DDD” could reflect the economic burden for patients.

(2) $$\begin{array}{c} \text{Average costs}=\frac{\text{Total costs}}{\text{number of hospitals}\times 12\text{ months}},\\ \text{Average costs per DDD}=\frac{\text{Average costs }}{\text{Average volume }}. \end{array}$$

### 2.5. Statistical Analysis

In this study, the variations in an index of the two groups before and after policy intervention are calculated to reflect the net effect of county public hospital reform. The differences lie in the cluster-level summaries of the two groups. A difference-in-differences (DID) methodology was used to compare pre- and postreform changes between the intervention group and the control group. For each group, the incremental effect of the policy intervention was computed as the differences between the pre- and post-implementation of policy intervention, and then, the incremental effects were compared between the intervention and control groups to estimate the net effect of the policy intervention [50]. The DID regression model is constructed as follows:

(3) $$\begin{array}{c} Y_{ijt}=\alpha_{0}+\beta \times {\text{time}}_{ijt}\times {\text{group}}_{ijt}+\gamma \times X_{it}+\varepsilon_{ijt}, \end{array}$$

where *i* indicates the county public hospital, *j* indicates the specific antibiotic, and *t* indicates the month. *Y* represents main outcomes, either the average volume, costs, or costs per DDD; time_*ijt*_ is time dummy variable, where prereform (January 2012 to December 2012) is 0 and postreform (January 2013 to December 2013) is 1; group_*ijt*_ is treatment dummy variable, where the intervention group is 1 while the control group is 0, where time_*ijt*_ × group_*ijt*_ is an interaction term between time dummy variable and treatment dummy variable, and the key coefficient *β* reflects the net effect of the county public hospital reform policy; *X*_*it*_ is a series of covariates, including permanent population, per capita GDP, average annual income for urban residents, and average annual income for rural residents; *ε*_*ijt*_ is the residual error.

As some outcomes do not follow normal distributions, we employed generalized linear regressions to perform DID analysis. The modified Park test was used to test the family distribution and Box–Cox test to test the link function [39]. Based on the results shown in Table 3, we used gamma distribution and square root link for the average volume of total, nonrestricted, and injectable antibiotics, for average costs per DDD of injectable antibiotics, and for costs of total, restricted, and injectable antibiotics; we used gamma distribution and log link for average volume of controlled and oral antibiotics, for average costs per DDD of oral antibiotics, and for costs of controlled antibiotics; we used Poisson distribution and square root link for average volume of restricted antibiotics, for average costs per DDD of total and controlled antibiotics, and for costs of restricted antibiotics; we used Poisson distribution and log link for costs of oral antibiotics; we used Gaussian distribution and square root link for average costs per DDD of restricted antibiotics while log link for average costs per DDD of nonrestricted antibiotics.

One important premise of the DID approach is that the intervention group and the control group should meet the common-trend hypothesis, indicating that no systematic differences in the trends can be observed between two groups before the implementation of policy [51]. Usually, it is not easy to meet the common-trend hypothesis; therefore, we used time series analysis to test the hypothesis. The placebo test in the DID method was used to test the robustness of the findings. For example, if the policy was implemented in period *t*, we could assume the policy implemented in *t* − 1 or *t* − 2 or *t* + 1 and other periods instead of the actual period *t*, and then, a DID analysis was used to assess the differences between two groups. We assumed the policy was implemented in July 2012 instead of the actual Jan 2013, and we need to estimate the differences of two groups between Jan 2012 and December 2012. The rationale of the test is that if the differences prior to Jan 2013 were driven by selection bias due to some unobserved, time-varying heterogeneities, then these heterogeneities would lead to a placebo result in the truncated sample [52, 53]. The placebo test in this study was prevalent in econometrics, and many studies focused on the test for robustness of findings [16, 54–58]. If the results of placebo test are significant, the differences are likely to be caused by selection bias arising from some unobserved heterogeneities.

## 3. Results

### 3.1. Antibiotic Consumption and Costs before and after the Implementation of County Public Hospital Reform

On average, 1711.9 thousand RMB was spent on the procurement of antibiotics per hospital per month throughout the study period: 1730.5 thousand RMB in the intervention group for 41.43 thousand DDDs and 1579.5 thousand RMB in the control group for 35.60 thousand DDDs in the prereform period; 1860.2 thousand RMB in the intervention group for 39.08 thousand DDDs and 1732.5 thousand RMB in the control group for 39.21 thousand DDDs in the postreform period. The costs of injectable antibiotics accounted for approximately 87% of overall costs in both groups. The majority of procured antibiotics were nonrestricted (about 64% DDDs) and oral antibiotics (about 63%) regardless of groups. The average costs per DDD of controlled antibiotics (range 235.6-289.8 RMB) were the highest, followed by restricted (range 82.53-94.25 RMB) and injectable (range 97.84-108.45 RMB) antibiotics. The average costs per DDD of nonrestricted (range 18.89-22.82 RMB) and oral antibiotics (range 8.80-9.16 RMB) were much cheaper than the others.

As shown in Table 3, the average volume and costs increased in the control group while they decreased in the intervention group after the implementation of county public hospital reform: a 10.14% increase in DDDs and a 9.69% increase in costs in each hospital in the control group compared with a 7.50% increase in costs while a 5.67% decrease in DDDs in each hospital in the intervention group. For the intervention group, the average volume of restricted antibiotics and injectable antibiotics increased by 24.12% and 2.75% while the costs increased by 19.01% and 9.09%, respectively. However, the average costs per DDD of antibiotics increased in both groups after the implementation of the reform: an increase of 0.18 RMB in the control group compared with an increase of 4.92 RMB in each hospital in the intervention group.

### 3.2. Effects of County Public Hospital Reforms on the Consumption and Costs of Procured Antibiotics

The results of the effects on the outcomes of county public hospital reform are shown in Tables 4 and 5. The estimation of coefficient represents the interaction term between the time variable and group variable, indicating the actual effect of the reform. The DID results showed that the reform had a negative impact on average volume (*p* = 0.005) and costs (*p* = 0.001) of nonrestricted antibiotics. In addition, the implementation of the reform was associated with a 9.88% reduction in average volume (*p* = 0.031) and a 2.75 reduction (*p* = 0.043) in average costs of procured oral antibiotics. The reform also related with an increase in average costs per DDD of total antibiotics (*p* = 0.049). Although the average volume and costs of procured restricted and injectable antibiotics increased after the reform, there is no statistical significance.

### 3.3. Placebo Test and Time Series Analysis

**Figure 1:**
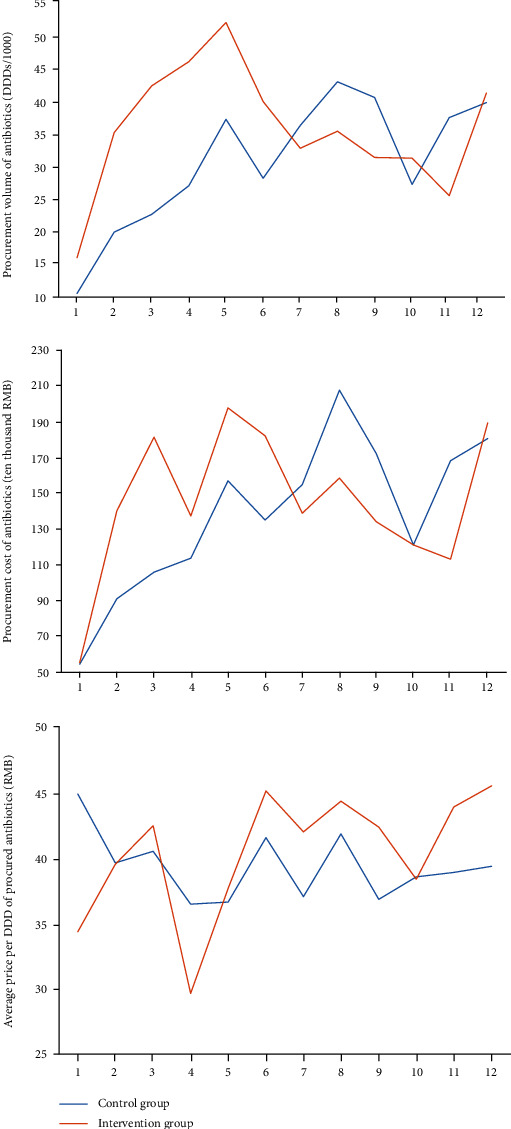
Average volume, costs, costs per DDD of procured antibiotics per hospital per month. Note. *Average* *volume* (*DDDs*/1000) = Total DDDs/number of hospitals × 12 months; *Average* *costs* = Total costs/number of hospitals × 12 months; *Average* *costs* *per* *DDD* = Average costs /Average volume .

Table 6 shows that the coefficient estimates of placebo test were statistically insignificant, indicating that the premise of common trend in two groups was satisfied. Additionally, the results also suggested the differences were unlikely to be caused by some unobserved heterogeneities. The secular trends of outcomes of the two groups before January 2013 were performed using time series analysis (Figure 1). There were no differences between the two groups in secular trends of average volume, costs, and costs per DDD (*p* = 0.226, *p* = 0.172, and *p* = 0.495) of antibiotics when all the county public hospitals had not implemented the reform.

## 4. Discussion

By using a quasiexperiment design, the main objective of this study was to focus on the impacts of county public hospital reform on the consumption and costs of procured antibiotics in Jiangsu, Eastern China. With a large sample from the eastern region of China, the findings of this quantitative study, together with the previously conducted study in Hubei, provide a comprehensive report of effects of county public hospital reform on the antibiotic use in China.

In general, there was a decreasing trend in antibiotic consumption in the 37 county public hospitals of the intervention group after the reform. Conversely, an increasing trend in antibiotic consumption was observed in the 62 county public hospitals of the control group after the reform. Meanwhile, there was an increasing trend in antibiotic expenditures in all county public hospitals after the reform. The DID results suggest the reform was associated with a reduction in the consumption of procured total antibiotics and costs of the nonrestricted and oral antibiotics. In contrast, the reform was linked with an increase in the costs per DDD of total antibiotics. Although the reform could exert some positive changes on antibiotic consumption, a nonsignificant decrease was observed in the costs of total antibiotics in the 37 county public hospitals of the intervention group. However, the structure of drug usage could be changed by the reform. The decreasing trend in antibiotic consumption by the reform found in this study means the cost per prescription has the possibility to reduce. This finding was consistent with previous studies that claimed the zero-markup reform has reduced the average cost per prescription [35].

Regarding the total antibiotic consumption, it is inconsistent with the findings in Hubei where there was an increasing trend in procurement volumes and expenditures on antibiotics after the reform [16]. Consistent with our finding, the reform also led to a significant increase in costs of parenteral antibiotics in Hubei [16].

To promote the appropriate use of antibiotics, China has issued a series of policies and regulations in the past decade. The antibacterial stewardship policy was implemented in medical institutions nationwide, but its administrative color was strong. This policy required all hospitals to establish a hierarchical management system for clinical antibiotic use, which could pose the same impacts on the antibiotic use of the intervention group and the control group simultaneously. Thus, due to DID analysis, this policy has little effect on the results of this study. Furthermore, the administrative antibacterial stewardship policy could not cut off the relationship between doctors' benefits and drug revenues. The doctors' mechanisms of behavior cannot be changed by the antibacterial stewardship policy.

The potential reason was that a significant rise in the costs per DDD of total antibiotics after reform was mainly attributed to the growth of the consumption and costs of restricted antibiotics and parenteral formulations. It is noted that although the consumption of nonrestricted and oral antibiotics with lower procurement unit prices had declined, the consumption of expensive restricted and injectable antibiotics kept rising instead. The procurement unit prices of parenteral antibiotics were much higher than those of oral antibiotic. Generally speaking, most of restricted antibiotics are costly antibacterials, which include meropenem, imipenem, cefoperazone/sulbactam, ceftazidime, parenteral ciprofloxacin, and vancomycin [59]. Consequently, the significant reduction in the expenditures of nonrestricted and oral antibiotics should be easily offset by the unexpected increase in the consumption and costs of restricted and injectable antibiotics. Even so, our finding suggested that the reform could play a role in reducing the abuse of some antibiotics. However, it also showed that the physicians of county public hospitals prescribed greater amount of expensive antibiotics after the reform. This is contrary to the previous study regarding antibiotic prescriptions in county hospitals, which showed that far less expensive antibiotics were prescribed in county hospitals after removing the markup of drugs [60].

Our findings suggested that there are causal chains between the four key components in reform and the main outcomes. First, zero markup on the sale of medicines exerted a positive influence in the volume and cost of nonrestricted and oral antibiotics. It meant that the county public hospitals could no longer profit from the sale of nonrestricted and oral antibiotics with lower procurement unit prices. However, under the reformed fee schedules, they could charge a fee for drug-associated services, such as injectable antibiotics, which led to a significant increase in the cost of injectable antibiotics. Second, although the government increased financial subsidies to hospitals to compensate for their income loss, the county public hospitals in Jiangsu still faced the challenge of serious financial crisis. Even in the developed region of Jiangsu province, the government budget for the pilot county public hospitals as a share of hospital revenues increased only slightly from 4.48% in 2013 to 7.67% in 2014 [35]. The incomes of medical workers are linked to hospital revenues. In order to compensate for the income loss due to zero markup of drug sales, the volume of drug-associated services with parenteral antibiotics increased markedly. It may be counterproductive that the financial subsidies could not play their due roles in the reform. Trading off and taking turns, when the volume and cost of injections increased, the oral antibiotics decreased proportionately. Third, although the government removed the markup of drugs, the link between sales of prescribed medicines and income of doctors had not decoupled completely, which likely led to a distortion in the prescription of expensive medicines in China [60]. Economic motivation still plays a key role in the prescribing practice [61]. The reform measures do not have the necessary prevention function of the improper sales activities of pharmaceutical companies. It is commonly argued that the volume-based contingent commissions offered by pharmaceutical companies provide an incentive for doctors to prescribe unnecessary expensive antibiotics. A study conducted in Beijing also observed the public hospitals increased use of expensive medicines after removing the profits of drugs [62]. Unfortunately, as no detailed guidelines were provided in the policy documents, the payment reform of health insurance did not achieve the anticipated goal, even little action of payment mechanism reform was put into practice. Therefore, it has little effect on the promotional activities of pharmaceutical companies. In conclusion, considering the different impacts of the four components of county public hospital reform on antibiotic use, only the volume and cost of nonrestricted and oral antibiotics decreased significantly.

In addition, several possible explanations can be reasonable for the findings. First, due to inadequate government subsidies, it was difficult for the hospital to compensate for the loss of profit from the drug sales under the background of zero-markup drug reform [63, 64]. Evidence showed that the financial support from the government accounted for about 10% of public hospital revenues [32]. Due to the shortage of financial subsidies, a close association between hospital revenues and hospital employees' income could contribute to an increase in volume of services. For example, an empirical study in Tianjin observed the implementation of county public hospital reform led to a substantial increase in the costs of medical service [51]. On the one hand, more hospital admissions were encouraged by the public hospital administrator [65–67], even though it was not necessary for some patients to receive hospitalization service. The county public hospitals could charge higher service fees for hospitalized patients to compensate for the loss of revenues. In addition, it increases chances for hospitals to provide services for parenteral administration of drugs [68]. On the other hand, fees for therapeutic service were the other source of hospital revenues. Even though the profits of medicines were removed, county public hospitals could increase charges for other services requiring professional skills, such as intravenous infusion, and induce demand for these services from patients. Another empirical study conducted in Hubei indicated that the out-of-pocket spending of patients increased after the implementation of the zero-markup drug reform, and the largest increase was from therapeutic service [39].

Second, the affordability of medicines has been improved by the zero-markup reform on the drug sales, which may increase the probability of patients requesting more prescriptions with perceived significant curative effect. Compared with cost-effective oral drugs, injections are considered as the stronger, faster, and more high-quality medicines for treatment [29]. Therefore, the prevalence of injection use may be associated with its perceived great efficacy. In China, patients have a deep-rooted misconception that the intravenous infusion is more effective than oral drugs [69], which led to continued demand for injections from patients. The misconception is caused by low Chinese health literacy [70], and thus, patients with insufficient health education prefer to choose injections as priority treatment. Moreover, though WHO recommends that the injectable antibiotics should be prescribed only when necessary [71], patients without proper indications which require prescriptions for antibiotics are common [72]. For example, Reynolds and McKee showed that patients with a common cold would request treatment of injectable antibiotics if the clinical symptoms persisted after the treatment of oral medicines [29]. Kutsogiannis et al. also indicated that under the pressure from patients' parents, most physicians still prescribed injections though no clinical indication for parenteral treatment was found [73]. Therefore, the unnecessary demand from patients may lead to an increase in the consumption and costs of injectable antibiotics. Meanwhile, there is another common misconception in China that the expensive medicines are more effective than the cheap ones. Thus, this may be a possible explanation for the decrease in consumption and costs of oral and nonrestricted antibiotics while an increase in injectable and restricted antibiotics.

There are several policy implications in this study. First, the effects of public hospital reform may vary between procured antibiotics and other medicines. Unlike other medicines, AMR was a serious public health problem in China, which required more specific and targeted measures for county public hospitals to improve the rational use of antibiotics. However, some studies indicated that removing the economic incentives had a negative effect on medical service quality [26]. Therefore, the government should establish a dynamic and reasonable financial compensation system for the loss of revenues, such as increasing the financial subsidies for the public hospital's operation. Second, reducing the economic incentives for doctors could be the most powerful way to curb overuse of antibiotics in the public hospital reform. The government should develop strategies to cut off the relationship between doctors' benefits and drug revenues. A dynamic incentive mechanism of cost-effective medicine should be strengthened to guarantee access to affordable medicines. Finally, the institutionalized antimicrobial stewardship (AMS) programs should be implemented in county public hospitals to curb antibiotic abuse, with particular emphasis placed on the use of controlled and restricted antibiotics.

Our study had several strengths. First, the study avoided the sampling bias as the procured data were derived from all of the county public hospitals in Jiangsu province. Second, the DID method can control the confounding influences of dependent variables and solve the endogeneity problem and the placebo test can ensure that the results of DID estimation are unbiased. Nevertheless, several limitations should be acknowledged. Firstly, the procured data could not measure the antibiotic use at the patient level, which prevented us from evaluating the impacts of the reform on the patients' appropriateness of antibiotic use. Future studies could combine individual patient-level data from health insurance system to explore deep-rooted and underlying problems. Secondly, as no separated data were recorded in the procurement system, we cannot calculate antibiotic use for outpatient and inpatient care separately. To shed light on quality use of antibiotics, further studies, using data from the prescription system, could evaluate the effects of reform on outpatient and inpatient care separately. Thirdly, due to the pricing policy issued from the National Development and Reform Commission, the price of some antibiotics changed during the study period, which could limit the implication of cost indicator. Fourth, there was statistical significance in the GDP per capita and the per capita disposable income of urban residents, which could lead to a bias between the two groups.

## 5. Conclusions

Under the background of county public hospital reform in Jiangsu, the profit margin on drug sales in public hospitals was reduced significantly. The reform is effective in reducing the consumption and costs of unrestricted and oral antibiotics. However, the reform could not change the funding system for public hospitals completely. It has failed to reduce the consumption and costs of expensive restricted and injectable antibiotics, leading to increased costs of diseases. It is critical that the health policy can deincentivize overuse of antibiotics at both hospital and individual physician's levels. The public hospital reform should enforce government financial support, improve hospital governance, optimize performance evaluation, and establish specialized management approach for antibiotic use.

## Figures and Tables

**Table 1:** Key components of county public hospital reform in Jiangsu province.

Key components	Before reform	After reform	Objectives
Zero-markup policy on the sale of drugs	Adding a 15% profit margin on the sale of drugs	Removing the markup on the sale of drugs	To cut off the economic incentives and improve affordability of medicines
Price adjustment policy on medical service	Keeping consultation fees low	Increasing fees for consultations and skilled services	To compensate for the loss of drug sales and optimize salary system for medical personnel
Finance subsidy policy on county public hospitals	Reducing the amount of the government subsidies to county public hospitals	Increasing the financial subsidies to county public hospitals	To compensate for the loss of drug sales
Payment reform of health insurance	A purely fee-for-service (FFS) system	A mixed payment system that included prospective payment methods	To control the rapid increasing of medical cost

**Table 2:** Characteristics of counties in which the hospitals included in the study are located.

County characteristics	Control group (*n* = 62)	Intervention group (*n* = 37)	*t*	*p* value
Population (ten thousand)	88.87 ± 31.92	96.22 ± 33.42	1.185	0.127
GDP per capita (RMB)	69912.75 ± 42898.00	88462.90 ± 32397.14	2.414	0.017
Per capita disposable income of urban residents (RMB)	29564.50 ± 8307.11	33899.36 ± 7264.85	2.815	0.006
Per capita disposable income of rural residents (RMB)	11870.06 ± 4926.92	12896.46 ± 3853.59	1.244	0.217

**Table 3:** The consumption and costs of procured antibiotics in county hospitals.

Outcomes	Total	Prereform (2012.1-2012.12)		Postreform (2013.1-2013.12)		Change rate (%)	
		Control group	Intervention group	Control group	Intervention group	Control group	Intervention group
Average volume (DDDs/1000) of procured antibiotics per hospital per month							
Total antibiotics	38.54	35.60	41.43	39.21	39.08	10.14	5.67^∗^
Nonrestricted	24.93 (64.69)	22.45 (63.06)	28.99 (69.97)	25.29 (63.50)	24.30 (62.18)	12.65	16.18^∗^
Restricted	12.57 (32.62)	11.66 (32.75)	11.36 (27.42)	13.12 (33.46)	14.10 (36.08)	12.52	24.12
Controlled	1.04 (2.70)	1.49 (4.19)	1.08 (2.61)	0.80 (2.04)	0.68 (1.74)	46.31^∗^	37.04^∗^
Oral	24.41 (63.34)	21.63 (60.76)	27.62 (66.67)	24.73 (63.07)	24.89 (63.69)	14.33	9.88^∗^
Injectable	14.13 (36.66)	13.97 (39.24)	13.81 (33.33)	14.48 (36.93)	14.19 (36.31)	3.65	2.75
Average costs (ten thousand RMB) of procured antibiotics per hospital per month							
Total antibiotics	171.19	157.95	173.05	173.25	186.02	9.69	7.50
Nonrestricted	45.20 (26.40)	40.01 (26.33)	46.06 (26.62)	50.27 (29.02)	44.10 (23.70)	25.64	4.26^∗^
Restricted	105.71 (61.75)	92.21 (58.38)	104.34 (60.29)	107.71 (62.17)	124.18 (66.76)	16.81	19.01
Controlled	20.28 (11.85)	25.73 (16.29)	22.65 (13.09)	15.27 (8.81)	17.74 (9.54)	40.65^∗^	21.68^∗^
Oral	21.60 (12.62)	19.57 (12.39)	23.25 (13.44)	21.83 (12.60)	22.61 (12.15)	11.55	2.75
Injectable	149.59 (87.38)	141.01 (87.61)	151.71 (86.56)	154.04 (87.40)	164.62 (87.85)	9.42	9.09
Average costs per DDD (RMB) of procured antibiotics per hospital per month							
Total antibiotics	47.96	49.03	43.61	49.21	48.53	0.37	11.28
Nonrestricted	21.26	21.04	18.89	22.82	21.35	8.46	13.02
Restricted	87.70	86.34	91.52	82.53	94.25	4.41^∗^	2.98
Controlled	263.23	235.60	279.60	264.90	289.80	12.44	3.65
Oral	8.98	9.16	8.80	8.94	8.93	2.40^∗^	1.48
Injectable	100.15	97.84	101.85	98.07	108.45	0.24	6.48

Note. Average volume (DDDs/1000) = Total DDDs/(number of hospitals × 12 months); Average costs = Total costs/(number of hospitals × 12 months); Average costs per DDD = Average costs/Average volume; ^∗^decreased.

**Table 4:** Results of modified Park tests and Box–Cox tests to identify family distribution and link function of outcome indicators.

Outcomes	Modified Park test	Family distribution	Box–Cox test	Link function
Average volume (DDDs/1000) of procured antibiotics per hospital per month				
Total antibiotics	2.15	Gamma	0.32	Square root
Nonrestricted	2.61	Gamma	0.28	Square root
Restricted	1.29	Poisson	0.30	Square root
Controlled	2.06	Gamma	0.15	Log
Oral	1.76	Gamma	0.25	Log
Injectable	2.79	Gamma	0.35	Square root
Average costs (ten thousand RMB) of procured antibiotics per hospital per month				
Total antibiotics	2.05	Gamma	0.31	Square root
Nonrestricted	1.03	Poisson	0.30	Square root
Restricted	2.22	Gamma	0.27	Square root
Controlled	2.08	Gamma	0.14	Log
Oral	0.91	Poisson	0.24	Log
Injectable	2.11	Gamma	0.31	Square root
Average costs per DDD (RMB) of procured antibiotics per hospital per month				
Total antibiotics	1.31	Poisson	0.61	Square root
Nonrestricted	0.28	Gaussian	0.20	Log
Restricted	0.32	Gaussian	0.70	Square root
Controlled	0.76	Poisson	0.55	Square root
Oral	2.92	Gamma	0.04	Log
Injectable	1.68	Gamma	0.50	Square root

Note. Average volume (DDDs/1000) = Total DDDs/(number of hospitals × 12 months); Average costs = Total costs/(number of hospitals × 12 months); Average costs per DDD = Average costs/Average volume.

**Table 5:** DID results from generalized linear regression analyses.

Outcomes	Coefficient	*p*	Robust SE	Goodness-of-fit tests		
				AIC	BIC	Pearson
Average volume (DDDs/1000) of procured antibiotics per hospital per month						
Total antibiotics	-0.314	0.173	0.230	9.228	-15082.770	1680.131
Nonrestricted	-0.561	**0.005**	0.201	8.347	-14970.870	2001.965
Restricted	0.009	0.955	0.158	14.420	6066.151	27403.665
Controlled	0.150	0.317	0.150	1.961	-9029.707	3574.243
Oral	-0.182	**0.031**	0.085	8.261	-14282.020	2068.528
Injectable	0.010	0.949	0.152	7.405	-14746.730	1794.867
Average costs (ten thousand RMB) of procured antibiotics per hospital per month						
Total antibiotics	-0.037	0.950	0.587	12.180	-14532.80	2220.684
Nonrestricted	-0.923	**0.001**	0.286	40.291	61137.93	92155.146
Restricted	0.100	0.852	0.534	11.396	-14302.96	2532.157
Controlled	0.274	0.065	0.148	7.838	-9451.301	3197.465
Oral	-0.190	**0.043**	0.094	21.560	20905.560	42292.498
Injectable	0.046	0.936	0.570	11.955	-14275.830	2326.866
Average costs per DDD (RMB) of procured antibiotics per hospital per month						
Total antibiotics	0.283	**0.049**	0.144	16.716	7846.753	23872.654
Nonrestricted	0.043	0.540	0.071	8.480	603,713.100	620,722.325
Restricted	0.314	0.164	0.226	10.672	5,444,966	5,461,653.573
Controlled	-0.629	0.147	0.433	73.618	96057.450	111,453.308
Oral	-0.023	0.583	0.041	6.489	-14114.360	379.906
Injectable	0.097	0.605	0.187	11.153	-16566.770	480.957

Note. Average volume (DDDs/1000) = Total DDDs/(number of hospitals × 12 months); Average costs = Total costs/(number of hospitals × 12 months); Average costs per DDD = Average costs/Average volume.

**Table 6:** Placebo tests of the data from January 2013 to December 2014.

Outcomes	Modified Park test	Box–Cox test	Coefficient	*p*	95% CI	Robust SE
Average volume (DDDs/1000) of procured antibiotics per hospital per month						
Total antibiotics	1.81	0.30	-0.474	0.142	(-1.106, 0.158)	0.323
Nonrestricted	2.09	0.29	-0.497	0.092	(-1.074, 0.080)	0.294
Restricted	1.85	0.28	-0.194	0.325	(-0.580, 0.192)	0.197
Controlled	1.10	0.13	-0.179	0.374	(-0.575, 0.216)	0.202
Oral	1.92	0.26	-0.423	0.144	(-0.992, 0.145)	0.290
Injectable	2.14	0.31	-0.076	0.714	(-0.483, 0.331)	0.208
Average costs (ten thousand RMB) of procured antibiotics per hospital per month						
Total antibiotics	2.32	0.28	0.053	0.945	(-1.463, 1.569)	0.774
Nonrestricted	1.82	0.26	-0.358	0.379	(-1.153, 0.438)	0.046
Restricted	2.27	0.25	0.065	0.923	(-1.261, 1.392)	0.677
Controlled	1.35	0.12	0.078	0.683	(-0.295, 0.450)	0.190
Oral	1.78	0.23	-0.167	0.118	(-0.399, 0.065)	0.159
Injectable	2.62	0.28	0.216	0.775	(-1.268, 1.701)	0.757
Average costs per DDD (RMB) of procured antibiotics per hospital per month						
Total antibiotics	2.29	0.32	0.134	0.071	(-0.011, 0.278)	0.074
Nonrestricted	3.40	0.16	0.199	0.043	(0.007, 0.391)	0.098
Restricted	1.70	0.33	0.395	0.149	(-0.141, 0.932)	0.274
Controlled	1.20	0.55	0.708	0.253	(-0.501, 1.923)	0.620
Oral	1.28	0.32	0.074	0.478	(-0.130, 0.278)	0.104
Injectable	0.89	0.61	0.129	0.566	(-0.313, 0.572)	0.226

Note. Average volume (DDDs/1000) = Total DDDs/(number of hospitals × 12 months); Average costs = Total costs/(number of hospitals × 12 months); Average costs per DDD = Average costs/Average volume.

## Data Availability

Data are available from the corresponding authors for researchers who meet the criteria for access to confidential data.
